# Clinician acquisition and retention of Motivational Interviewing skills: a two-and-a-half-year exploratory study

**DOI:** 10.1186/1747-597X-5-8

**Published:** 2010-05-13

**Authors:** Lisa Forsberg, Lars G Forsberg, Helena Lindqvist, Asgeir R Helgason

**Affiliations:** 1 Department of Clinical Neuroscience, Karolinska Institute, 171 76 Stockholm, Sweden; 2 Department of Public Health Sciences, Social Medicine, Karolinska Institute, Box 17070, 104 62 Stockholm, Sweden; 3 School of Health and Education, Reykjavik University, 103 Reykjavik, Iceland

## Abstract

**Background:**

Motivational interviewing (MI) is a collaborative, client-centred counselling style aimed at eliciting and strengthening clients' intrinsic motivation to change. There is strong research evidence supporting the efficacy of MI, notably in its application among alcohol and drug abuse populations. MI interventions in smoking cessation may yield modest but significant increases in quitting. The present study sought to assess the acquisition and retention of MI skills in counsellors at the Swedish National Tobacco Quitline.

**Methods:**

Three audio-recorded sessions from each of three counsellors were assessed using the Motivational Interviewing Treatment Integrity (MITI) Code Version 3.0 over 11 assessment periods at fixed intervals in a two-and-a-half year period during which counsellors received ongoing supervision.

**Results:**

The mean skill for all counsellors improved throughout the study period in most MITI variables. However, great variations in MI skill between counsellors were observed, as well as fluctuations in performance in counsellors over time.

**Conclusion:**

The present exploratory study covers a longer time period than most evaluations of MI training, and has several advantages with regard to study design. It may provide a basis for (larger sample) replication to test MI skill (as measured by the MITI) in relation to behaviour change in clients, to evaluate MI training, and to assess the acquisition and retention of MI skill over time. Difficulties in acquiring and retaining MI skill may raise the issue of a selection policy for MI training. Moreover, fluctuations in MI skill over time emphasise the greater importance of continuous feedback and supervision over initial MI training, and the need for the use of validated treatment integrity assessment instruments in ordinary clinical implementations of MI.

## Background

### Motivational Interviewing

Motivational interviewing (MI) is a collaborative, client-centred counselling style aimed at eliciting and strengthening clients' intrinsic motivation to change [1]. The principles of MI include cultivating discrepancies between the client's behaviour and goals, expressing empathy, eliciting self-motivational statements (for example, statements expressing reasons for change), non-confrontation of resistance and supporting self-efficacy [2]. There is strong research evidence supporting the efficacy of MI, notably in its application among alcohol and drug abuse populations [3], but also in a number of other behaviour changes [4], When MI is used as a prelude to other treatments, outcomes appear to have greater longevity and be more steadfast compared to when used as a stand-alone treatment [3]. Research into the efficacy of MI in smoking cessation has produced mixed results, although a recent systematic review (of randomised control trials comparing MI to brief advice, usual care, and self-help materials) found that MI interventions may yield modest but significant increases in quitting [5].

### Training of counsellors in MI: what we know

MI training ordinarily comprises a two to four-day workshop. Short lectures are interspersed with video demonstrations and exercises where participants can practice specific MI skills. In some settings, such as primary care, MI training time is often shorter, with training comprising as little as two hours [6].

Teaching of MI techniques in these workshops has been shown to influence counsellor behaviour [7]. A pilot study on the effects of a two-day MI training workshop on staff working within the probation services showed an increase in newly acquired MI skills after completion. However, MI skills were not maintained at three-month follow-up [8].

In one study, 140 well-educated and motivated participants were initially given *Motivational Interviewing: Preparing People for Change *[2], as well as seven demonstration videotapes [9]. The participants were randomly allocated to five groups, each undergoing different MI training. Measuring the degree of in-session change talk and counter-change talk in clients, only the participant group given a workshop, coded feedback, and telephone coaching on six submitted client sessions were able to procure significant increase in change talk and decrease in resistance after four months compared to baseline.

Demand for MI training is ever-increasing, and MI is continually applied to new settings and tested on different problems [4]. However, evidence of successful implementation of MI in ordinary clinical treatment settings is sparse [10,11]. MI, although ostensibly simple, appears to be rather a difficult method to implement to a high fidelity [10]. Therefore, how people acquire and retain MI skills and how the development of MI skills is best facilitated remain important areas of study [7,10].

### Evaluation of MI training

In order to have sufficient grounds for evaluation of the efficacy of MI, it is critical to assess to what extent counsellors responsible for delivering "MI treatment" are in fact using the method. However, treatment integrity has been an aspect overlooked in research into the efficacy of MI (in a recent systematic review, only 1 of 14 selected studies used a validated instrument to measure fidelity to MI principles) [5].

Several instruments aimed at facilitating monitoring, feedback, and research on MI skills have been developed [12-15]. One early instrument was the Motivational Interviewing Skills Code (MISC) [16]. The MISC measures both therapist and client behaviours, and the interaction between therapist and client and thus makes it possible to study the in-session MI process. However, consisting of more than 30 different variables, the MISC is labour-intensive and expensive to use. Further efforts lead to the development of a simpler instrument, the Motivational Interviewing Treatment Integrity (MITI) Code Version 2.0 [17]. In 2007, a revised and updated version of the MITI, Version 3.0, was introduced [18]. MITI's scope is more limited than the MISC, measuring therapist behaviour only.

MITI has proved a reliable tool for evaluating the use of MI [15,17,19], and has shown good discriminant validity with regard to in-session behaviour and MI skill development over time (entry-level competence and post-MI training) [10,20]. It has therefore been recommended as a way of evaluating MI training [7,14,15].

### Study aim

Knowledge on how to assist clinicians to learn MI is scare and evaluation periods are short [15]. The present study forms part of a larger project evaluating the effects of MI in smoking cessation among clients of the Swedish National Tobacco Quitline (SNTQ) and was approved by the Karolinska Institute Northern Research Ethics Committee (00-367). It sought to follow a group of counsellors during a longer period of training to see what outcome these efforts would have on their counselling behaviour.

The aims of the present exploratory study were to assess:

1. Development of MI skills in counsellors over time;

2. Which MI skills were most and least easily acquired by counsellors;

3. Time taken for counsellors to reach the recommended threshold for "Beginning Proficiency" suggested in the MITI 3.0 manual;

4. Efficacy of training/supervision efforts used for the purpose of the study in maintaining the level of MI proficiency over time.

## Methods

### Study setting

Telephone help lines (quitlines) are increasingly recognised as an effective [21,22], and cost-effective [23], form of intervention to treat tobacco smoking [23], which is one of the largest preventable public health problems [24,25]. SNTQ is a nationwide free of charge service operated by Stockholm County Council Health Service and funded by the Swedish Government. SNTQ has been in operation since May 1998. Throughout the study period three to four lines were in operation for approximately 51 hours per week. The present study forms part of a wider project evaluating the effects of adding MI to the standard tobacco counselling treatment protocol. Nine counsellors were allocated to the MI intervention group and nine counsellors were allocated to the standard treatment group. At the time of the study, there were 18 counsellors working at the SNTQ. Sessions with clients lasted on average 16 minutes.

### MI training and supervision of counsellors

The training of the nine SNTQ counsellors in the MI group was developed and conducted by a Motivational Interviewing Network of Trainers practitioner and clinical psychologist with considerable experience of MI training in various settings. Initial MI training, conducted in February 2003, comprised a two-day workshop focusing on practical exercise and also involving a combination of demonstrations, didactic and experiential activities. In total, 12 hours training was provided. Counsellors had access to the Swedish translation of *Motivational interviewing: Preparing people for change *and had been introduced to the MITI [26].

Initial training was followed by supervision in groups of four to five counsellors, supported by feedback based on MITI coding of audio-recorded sessions with actual SNTQ clients (counsellors chose which of their sessions were to be listened to in group supervision). Client consent was obtained before audio recording of sessions. During the first three months (Feb 2005-May 2005), counsellors were supervised every two weeks, with the focus on increasing the proportion of reflections (reflective listening skills), and decreasing the proportion of (unsolicited) advice and information giving. Over time training centred on increasing the proportion of complex reflections. Training goals were formulated and followed up on throughout the supervision period. During the first half of 2006 the emphasis was on increasing skill in the Empathy and MI Spirit variables, and in the latter half of 2006 particular attention was given to ability to recognise and reinforce change talk in clients. Throughout the whole study period, supervision aimed to prevent excessive and unsolicited advice and information giving by counsellors. Supervision was performed in 3-hour segments and totalled 84 hours.

### Criterion for study inclusion and counsellor background

Only counsellors who had three or more audio-recorded sessions for each of the 11 assessment periods were eligible for inclusion in the present study. Three of the nine counsellors allocated to the MI intervention met the criterion. Of the excluded six counsellors, two were excluded because they had other work assignments that did not allow for monitoring throughout the study period. The four remaining counsellors only had up to two recorded treatment sessions from two or more assessment periods. Work experience at the SNTQ ranged from 8-10 years. All counsellors included in the study had a background in traditional counselling (two were health pedagogues and one was a dental hygienist by profession) and were between 33 and 57 years of age.

### Treatment integrity

The MITI rating scheme rates practitioner behaviours. Coding involves the scoring of global variables, and counting the frequency of specific verbal behaviours [17,18]. In version 3.0 of the MITI, global ratings include MI Spirit (comprising three sub-variables: Evocation, Collaboration and Autonomy Support), Empathy, and Direction, which are rated on a five-point Likert-type scale ranging from 1 (low) to 5 (high). Detailed instructions on how to rate the global variables are provided in the MITI manual [18].

MITI behaviour frequency counts are Giving Information, MI Adherent and MI Non-Adherent statements, Questions (Closed and Open), and Reflections (Simple and Complex). Four calculated indices (Summary Scores) have been developed to measure MI skill: (1) Ratio of MI Adherent to MI Adherent and MI Non-Adherent statements; (2) Ratio of Reflections to Questions; (3) Ratio of Open Questions to Open and Closed Questions; and (4) Ratio of Complex Reflections to Simple and Complex Reflections [18].

MI Adherent statements are assigned for counsellor behaviours such as asking permission before giving advice or information; affirming clients or emphasising clients' control or autonomy; or supporting clients', for examples through expressing compassion. MI Adherent statements are assigned for counsellor behaviours such as advising without permission; confronting clients; or directing clients, for example through the use of orders, commands or imperatives [18]. Open questions have a wide range of possible answers, whereas closed questions are questions for which there is a particular answer, or to which only "yes" or "no" may be replied [18]. The client's response to the question does not affect its nature as Open or Closed. Simple Reflections convey understanding, yet add little or no meaning to the client's statements, whereas Complex Reflections add substantial meaning or emphasis to what the client has said [18].

For every global variable and index (Summary Score) there are recommended thresholds for "Beginning Proficiency" and "Competency" levels of MI skill respectively, based on the MITI coding instrument. These thresholds, suggested by the New Mexico University Research Group expert opinion, are however in need of further empirical testing [18].

In the present study, the coding was undertaken by two senior coders, from the Motivational Interviewing Coding Laboratory (MIC Lab) at the Karolinska Institute in Stockholm. MIC Lab coders participate in more than 40 hours initial training, in accordance with the recommendations of the University of New Mexico Research Group, followed by three-hour training sessions every fortnight. The two coders responsible for the coding of the material for the present study had worked at the MIC Lab since 2005 and 2006 respectively. The Swedish translation of MITI 3.0 was used for the study [19]. Recent research testing the validity of the MITI found that intra-class correlation (ICC) coefficients for MIC Lab coders trained in the Swedish translation of the MITI 3.0 demonstrated a significant improvement compared to ICC coefficients yielded in coding using MITI 2.0 [20], in statistical analysis where all variables were transformed to *z *data [20]. The authors concluded that MITI 3.0 appeared to be easier for coders to use reliably, especially with regard to the global variables, which are more specifically defined in MITI 3.0, while measuring the same constructs as MITI 2.0 [21].

Coder inter-rater reliability was high, with ICC coefficients ranging from 0.68 to 1.0 for both global variables and behaviour counts [20], thus ranging from "good" to "excellent" [27]. To reduce intra-rater and inter-rater variations, all material was coded during a four-week period in August-September 2007. Moreover, the same coder coded one counsellor for all his or her sessions. Coders were blind as to the dates of recorded material.

### Assessment periods

SNTQ counsellors were encouraged to audio-record treatment sessions at certain fixed intervals (assessment periods). Assessment periods ranged from two to six weeks, according to counsellor working hours. At each assessment period the three counsellors audio-recorded their first three sessions. Thus, for each assessment period, three audio-recorded sessions from each of the three SNTQ counsellors included in the study were treatment integrity assessed using the MITI 3.0. A mean score was calculated from the three different sessions for each of the counsellors, and for each of the MITI 3.0 variables at each assessment period. Therefore a particular MITI 3.0 score at a given point in time represents the mean of three different sessions.

Baseline assessment of counsellor MI skill was conducted between September and December 2004 (initial MI training was conducted in February 2003). Their MI skill as measured by the MITI was then assessed at 10 consecutive assessment periods at around six weeks interval until the study's end in early February 2007.

## Results

### MI skill as measured by MITI 3.0

**Figure 1:**
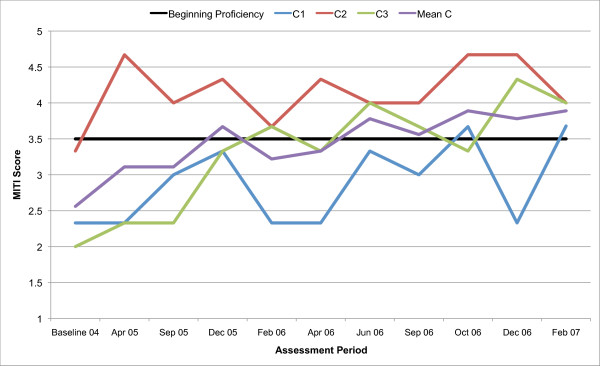
**MITI Empathy variable scores for counsellors C1, C2, C3**. Three coded sessions per assessment period for Sept/Nov 2004 (baseline) - Dec/Jan 2007, as well as mean for all counsellors.

**Figure 2:**
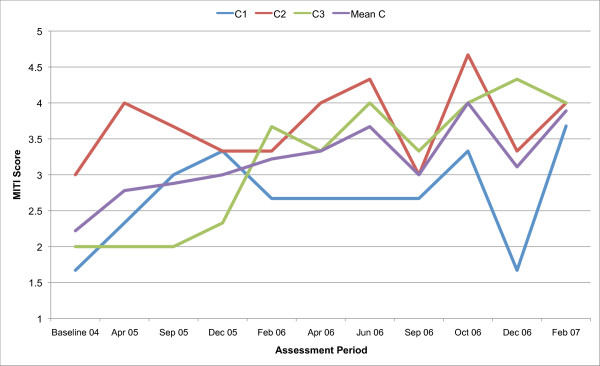
**MITI Evocation variable scores for counsellors C1, C2, C3**. Three coded sessions per assessment period for Sept/Nov 2004 (baseline) - Dec/Jan 2007, as well as mean for all counsellors.

**Figure 3:**
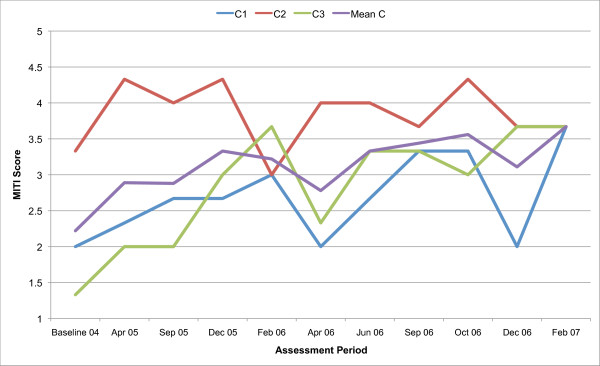
**MITI Collaboration variable scores for counsellors C1, C2, C3**. Three coded sessions per assessment period for Sept/Nov 2004 (baseline) - Dec/Jan 2007, as well as mean for all counsellors.

**Figure 4:**
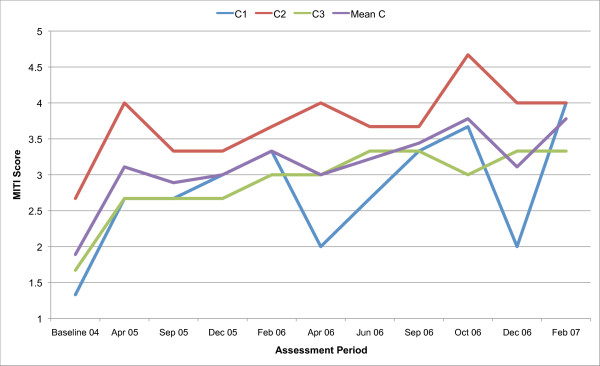
**MITI Autonomy Support variable scores for counsellors C1, C2, C3**. Three coded sessions per assessment period for Sept/Nov 2004 (baseline) - Dec/Jan 2007, as well as mean for all counsellors.

**Figure 5:**
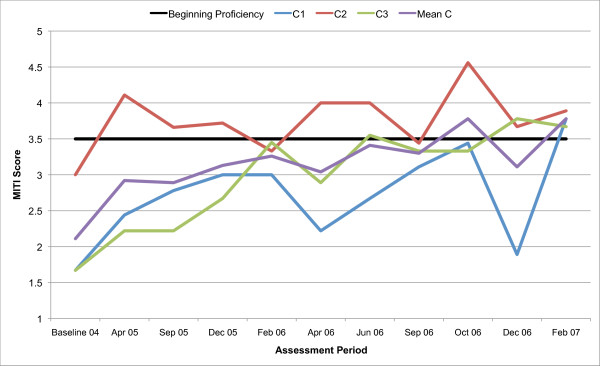
**MITI MI Spirit (mean Evocation, Collaboration, Autonomy Support) variable scores for counsellors C1, C2, C3**. Three coded sessions per assessment period for Sept/Nov 2004 (baseline) - Dec/Jan 2007, as well as mean for all counsellors.

**Figure 6:**
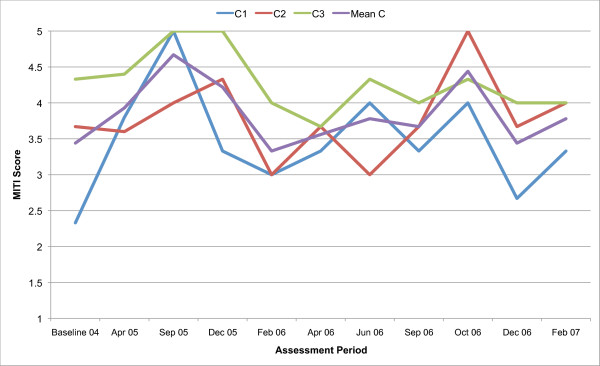
**MITI Direction variable scores for counsellors C1, C2, C3**. Three coded sessions per assessment period for Sept/Nov 2004 (baseline) - Dec/Jan 2007, as well as mean for all counsellors.

**Figure 7:**
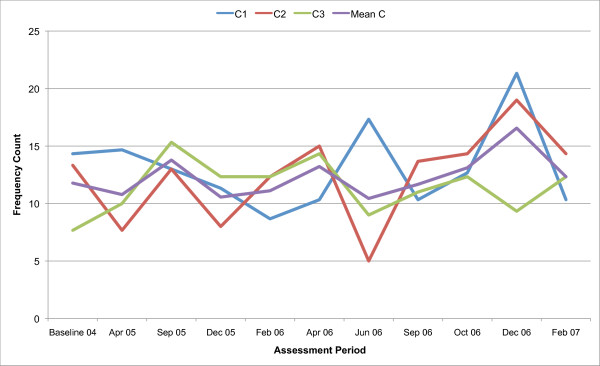
**MITI Giving Information behaviour count scores for counsellors C1, C2, C3**. Three coded sessions per assessment period for Sept/Nov 2004 (baseline) - Dec/Jan 2007, as well as mean for all counsellors.

**Figure 8:**
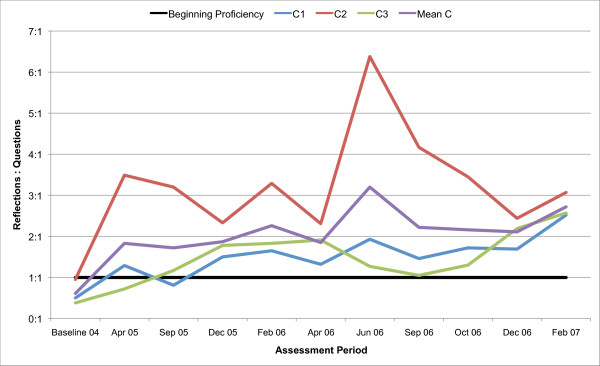
**MITI ratio Reflections to Questions index for counsellors C1, C2, C3**. Three coded sessions per assessment period for Sept/Nov 2004 (baseline) - Dec/Jan 2007, as well as mean for all counsellors.

**Figure 9:**
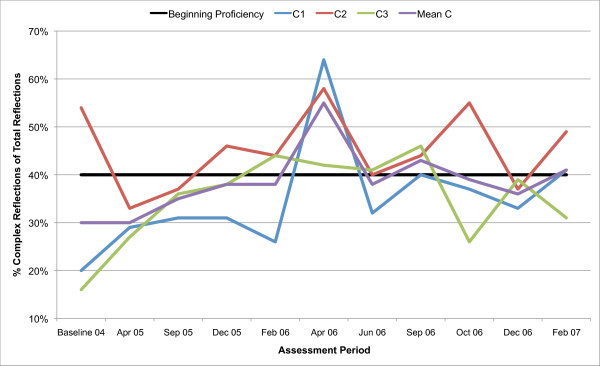
**MITI percentage Complex Reflections (Complex Reflections/Total Reflections) index for counsellors C1, C2, C3**. Three coded sessions per assessment period for Sept/Nov 2004 (baseline) - Dec/Jan 2007, as well as mean for all counsellors.

**Figure 10:**
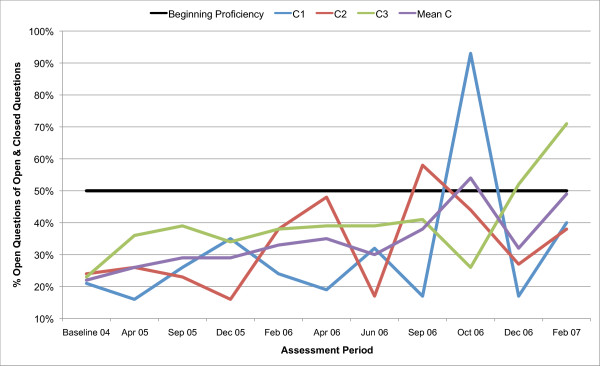
**MITI percentage Open Questions (Open Questions/Open + Closed Questions) index for counsellors C1, C2, C3**. Three coded sessions per assessment period for Sept/Nov 2004 (baseline) - Dec/Jan 2007, as well as mean for all counsellors.

**Figure 11:**
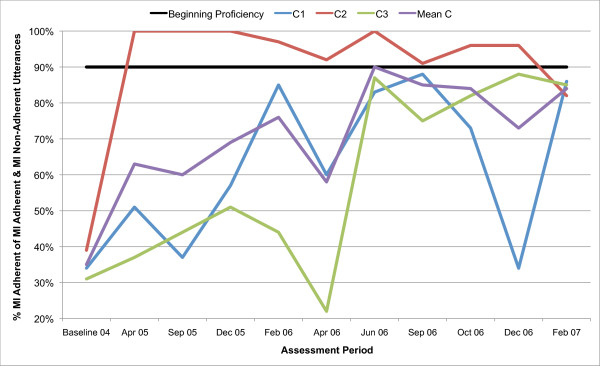
**MITI percentage MI Adherent (MI Adherent/MI Adherent + Non Adherent) index for counsellors C1, C2, C3**. Three coded sessions per assessment period for Sept/Nov 2004 (baseline) - Dec/Jan 2007, as well as mean for all counsellors.

All three counsellors in the present study are below the recommended threshold for Beginning Proficiency in all of the MITI variables at baseline assessment. Figures 1, 2, 3, 4, 5, 6, 7, 8, 9, 10 and  11 show each counsellor's MI skills and the mean score for all three counsellors, as measured by each of the MITI variables, throughout the two-and-a-half years of training and supervision. The threshold for Beginning Proficiency suggested in the MITI manual is indicated where applicable.

The Empathy variable (Figure 1) indicates to what extent the counsellor understands or makes an effort to grasp the client's perspective. Approximately 12 months post baseline, the mean for all three counsellors coincides with the MITI Manual's recommended threshold for Beginning Proficiency. There are great fluctuations in all counsellor Empathy scores and in counsellor 1 (C1) in particular. C1 remains below the recommended threshold for Beginning Proficiency until October 2006, almost two years post baseline. The most notable improvement is seen in counsellor 3 (C3). In counsellor 2 (C2) there is a rapid increase in skill post baseline, and retention of skill above the recommended threshold for Beginning Proficiency throughout the study period.

The Evocation scale (Figure 2) measures the extent to which the counsellor conveys an understanding that motivation to change, and the ability to move toward change, reside within the client, for example, by making efforts to elicit change talk. There are great variations between counsellors and fluctuations in counsellor performance over time. The mean for all three counsellors is continuously improving throughout the study period.

The Collaboration scale (Figure 3) measures the extent to which the clinician behaves as if the interview is occurring between two equal parties, both of whom possess knowledge that might be useful with regard to the problem under consideration. The development illustrated in the Collaboration variable is similar to that in Evocation. The mean shows an increase in Collaboration, but there are variations across counsellors and in counsellors over time.

The Autonomy Support variable (Figure 4) measures the extent to which the clinician supports and actively fosters client perception of choice, as opposed to attempting to control the client's behaviour or choices. There is an increase in MI skill in the Autonomy Support variable in all counsellors. There is a continuous improvement in skill throughout the study period in C3. The greatest fluctuations in demonstrated skill in the Autonomy Support variable are seen in C1. C2, rapidly increases in skill post baseline, remaining above the mean throughout the study period.

The mean of the Evocation, Collaboration and Autonomy Support variables gives the MI Spirit (Figure 5). The MI Spirit variable demonstrates a gradual increase in MI skill, but fluctuations are great. The mean for all three counsellors remains below the recommended threshold for Beginning Proficiency until October 2006, almost two years post baseline. The acquisition of MI Spirit skills appears to take considerably longer than, for instance, Empathy skills.

The Direction variable (Figure 6) measures the extent to which clinicians maintain appropriate focus on issues directly relevant to the specific target behaviour(s) that constitute the focal point for the session. The scores for this variable remained roughly constant for all counsellors throughout the study.

The Giving Information category (Figure 7) is used when the interviewer gives information, educates, provides feedback or discloses personal information, as well as when the interviewer gives an opinion, without advising. In this variable there is little change of note, neither over time nor across counsellors.

One behaviour count index that shows considerable variation across counsellors is the Ratio of Reflections to Questions (Figure 8). C2 remains constantly (and at times considerably) higher than the other counsellors. Nonetheless, after approximately six months, all counsellors demonstrate use of as many Reflections as Questions in their sessions.

In the percentage of Complex Reflections as a proportion all Reflections index (Figure 9), the mean remains below the recommended threshold for Beginning Proficiency until 15 months after the initial MI training of counsellors. There are great fluctuations in the use of Complex Reflections.

In the percentage of Open Questions as a proportion of Open and Closed Questions index (Figure 10), the mean remains below the recommended threshold for Beginning Proficiency until 20 months after initial MI training, and the ability to use Open Questions rather than Closed Questions is not retained after October 2006.

The percentage of MI Adherent utterances as a proportion of MI Adherent and MI Non-Adherent utterances index (Figure 11) is used to capture particular counsellor behaviours that are consistent with the MI approach, such as asking permission before giving advice or information or affirming the client. There are again considerable differences across counsellors. In C2, there is a rapid increase in skill demonstrated post baseline, performance thereafter remaining stable and above the recommended threshold for Beginning Proficiency up until the last assessment period. C1 and C3 improve steadily, but remain below the recommended threshold for Beginning Proficiency for the duration of the two-and-a-half year study period.

## Discussion

Only three counsellors had 3 or more audio-recorded sessions for each of the 11 assessment periods and were thus eligible for inclusion in the study. This factor severely limits the ability to draw conclusions based on the results. However, including counsellors with fewer recorded sessions from each assessment period would have made the MITI rating of counsellors' performance too vulnerable to difficulties experienced in individual sessions, such as particularly "difficult" clients, and compromised the study's usefulness as a model for large scale replication.

The findings of the present study suggest that in spite of its theoretical simplicity not all practitioners necessarily learn MI easily. Any successful implementation in a naturalistic clinical setting requires ongoing supervision of counsellors, including feedback and monitoring clinical practice. These findings are in line with previous research that sought to evaluate MI training and supervision interventions [9-11].

Like several other studies on MI training using objective outcome measures, the present study finds improvements in MI skill, for example, an increased use of reflections, after the initial workshop [7-9,28,29]. In a recent review only 1 out of 11 studies reported no significant difference post training relating to MI skill [7]. Moreover, our findings show that counsellors improved their MI skill compared to baseline level of skill in most of the MITI variables, though some variables took longer to improve. Most interestingly, the MITI variables continuously and steadily improved over the two-and-a-half year study period. From a client point of view, this means that the counsellor sessions were on average conducted more skilfully each assessment period.

Although the mean for the three counsellors included steadily improved throughout the study period, there were great variations in skill among counsellors and in learning time. There were no differences in background of counsellors included in the study that might explain the variance in acquisition of MI skill and performance over time. Literature in the field has found little or no correlation between the level of education or degree of work experience and positive outcomes, although variations in outcome have been found across counsellors [30-32]. However, none of this helps to explain the causes of particular counsellors' skill in certain variables.

According to counsellor comments, reduced in-session MI performance might depend on having "a bad day", tiredness and personal issues that negatively affect the ability to maintain appropriate focus, external disturbances of counsellor sessions in the workplace, and variations in difficulty of client problems. Such factors may account for some of the variations, both across counsellors and in counsellors over time. Moreover, differences in acquisition of MI skills could relate to the degree of motivation or readiness to change in counsellors. The study design might be improved by including an independent measure of counsellor readiness to change, or motivation to learn MI. More research is needed into possible causes for differences in skill and outcome in clients across clinicians.

The variability in acquisition and performance of MI skills, and the fact that performance within counsellors for specific skills varies over time, highlight the challenges facing those who wish to implement MI in real-world settings. The differences in ease of learning and learning time are of sufficient significance to raise the issue of a selection procedure for MI training (as has been suggested in the literature) [31,33], as well as rendering deeper consideration of training and supervision methods necessary. It may be that organisations wishing to implement MI would better apply their resources to ongoing supervision and treatment integrity assessment of MI practice, as opposed to only investing in costly MI workshop training - or at least develop a programme for continuous development of MI skill.

With regard to which MI skills were most and least easily acquired by counsellors, the Ratio of Reflections to Questions demonstrates a rapid improvement, suggesting that reflective listening may not be difficult for counsellors to learn. After approximately six months, the mean for the nine sessions coincided with the MITI Manual's recommended threshold for Beginning Proficiency. This is encouraging, as use of Reflections contributes to Empathy, which is thought to set a necessary stage for MI sessions and has been shown to be predictive of outcome [34,35]. From a pedagogic viewpoint, this finding favours MI training beginning with reflection or "OARS" (open questions, affirmations, reflections, summaries) type skills, which were the focus of all training schemes reviewed by Madson et al [7].

In the present study, it took two-and-a-half years for all three counsellors to reach the recommended threshold for Beginning Proficiency in the MI Spirit variables, which have also been found to have bearing on outcome [3]. MI training might benefit from greater concentration on skills associated with MI Spirit, such as eliciting change talk, by adding training designed to meet these needs after the initial workshop. Another finding, however, was that there were great variations in MI Spirit skill acquisition across counsellors, with C2 reaching Beginning Proficiency after a few months, and C1 only reaching Beginning Proficiency after two-and-a-half years of practice and supervision. This suggests that the ability to learn to use some MI techniques will vary considerably across potential MI practitioners.

The MITI Direction variable was the only of the global variables that remained roughly constant for all counsellors throughout the study. This is consistent with the backgrounds of the counsellors included in the study. All counsellors included were highly experienced in ordinary smoking cessation counselling. Unlike the other of the global MITI variables, a high score for Direction does not necessarily reflect a better use of MI, although the reverse is true, that is, in order for a session to reflect good use of MI, it must have a high Direction rating [2].

SNTQ counsellors have a manual comprising a number of Closed Questions, concerning, for example, client tobacco consumption and smoking patterns, which might have affected the proportion of Open Questions to Open and Closed Questions index negatively. Moreover, coders did not code counsellor questions on practical issues such as whether clients wanted material sent to them, or their contact details, since some counsellors included this as part of the session whereas others did not.

The data represents counsellor performance as acquired and retained over an extended period of time, in an ordinary clinical setting of the type where MI might be ordinarily implemented, thus providing a view of MI that is substantially different from typical RCTs demonstrating its efficacy. The great variation of MI skill in counsellors who have undertaken the same amount of training and supervision suggests that treatment integrity is crucial, in research as well as in ordinary clinical practice. In research aiming to accurately assess the efficacy of MI, the first step must be to ensure that practitioners responsible for delivering the treatment are indeed using MI (or a treatment which bears sufficient similarity to MI). The present findings suggest that attendance of initial MI training is not a good measure of MI skill. In a recent review of efficacy of MI in smoking cessation counselling, only 1 of 14 selected studies used a validated treatment integrity instrument [5]. Failure to account for the extent to which the proposed treatment intervention has been successfully implemented, through treatment integrity assessment, risks discrediting the treatment method itself, whereas the absence of positive outcome may instead be attributable to failed implementation. It is submitted that the use of MI in ordinary clinical practice ought to be subject to (at least) periodic assessment of treatment integrity using a validated instrument. Results of such assessment would not only serve as a trigger for "refresher" sessions where MI was not successfully implemented, but also provide a valuable learning tool for continued development of MI skills. Treatment integrity assessment would also help to ensure that clients receiving purported MI treatment actually receive that to which they consent.

One possible limitation is the nature of the SNTQ setting (self-initiated contact) that suggests considerable readiness for change among clients. However, it has been suggested that if a client is ready for change, the use of MI may stifle their desire to change [2]. It is possible that this is common in SNTQ clients, and identified by (some) SNTQ counsellors. Consequently, for clients who no longer display resistance or ambivalence towards smoking cessation, some counsellors may switch their approach to cognitive behavioural therapy inspired counselling. Such in-session behaviour would result in lower MITI scores, but not necessarily negatively influence outcome. To this extent, the validity of MITI is limited insofar as it is not constructed to capture complex therapist competence manifested in intentional and strategic use of MI [13], therefore potentially underestimating practitioner skill.

In spite of the limitations resulting from the small number of included counsellors, the present study has several important advantages with regard to design. The authors are not aware of any other studies where MI training has been followed by continual supervision and monitoring of counsellor MI skill in an ordinary clinical setting over as long a time period as the present study. Most other studies into the acquisition and retention of MI skills are limited to workshop format training efforts and pre- or post-training evaluations. Only 3 studies of 27 included in a recent systematic review by Madson et al. covered workshop and supervision [7], and only 1 described coaching after initial MI training [9]. This is despite the suggestion that a workshop-only format, although yielding improvements in knowledge and confidence, tends not to achieve competency or longevity in all MI variables [36,37]. Given these findings, and the considerable resources spent on attempts at en masse implementation of MI using workshop-only formats, studies covering longer time periods that examine training and supervision interventions in relation to acquisition and retention of MI skills in participants are essential, and the present study may provide a blueprint for future large scale research.

The study is one of the first to provide empirical data relating to Miller and Moyers' "Eight Stages in Learning Motivational Interviewing" [38]. However, contrary to what is suggested by Miller and Moyers, the results suggest that the OARS skills that form part of the second stage in learning MI are the skills that counsellors exhibit first in their clinical practice. The present study's design would be suitable for large sample replication to test the authors' hypotheses. Such a replication study has the potential to influence the MI training literature in an important manner. Similarly, the present study's design might serve as a basis for (large sample) replication to empirically test the recommended thresholds for Beginning Proficiency suggested in the MITI 3.0 manual, if outcome measures are related to MI skill.

## Abbreviations

**C1**: Counsellor 1; **C2**: Counsellor 2; **C3**: Counsellor 3; **ICC**: Intra-class correlation; **MI**: Motivational interviewing; **MIC Lab**: Motivational Interviewing Coding Laboratory; **MITI**: Motivational interviewing treatment integrity; **MISC**: Motivational Interviewing Skills Code; **OARS**: Open questions, affirmations, reflections, summaries; **RCT**: Randomised Controlled Trial; **SNTQ**: Swedish National Tobacco Quitline.

## Competing interests

The authors declare that they have no competing interests.

## Authors' contributions

LF drafted the manuscript, and made substantial contributions to the interpretation and analysis of results. LGF contributed to the conception and design of the study, in addition to the interpretation of the data and the initial drafting of the manuscript. HL contributed to the statistical analysis of data with regards to tobacco abstinence in clients, and made helpful comments on previous drafts of the manuscript. AH contributed with the material from the SNTQ, in addition to helpful comments on previous drafts of the manuscript. All authors approved the version to be published.
